# Bacterial Foraging Optimization –Genetic Algorithm for Multiple Sequence Alignment with Multi-Objectives

**DOI:** 10.1038/s41598-017-09499-1

**Published:** 2017-08-18

**Authors:** P. Manikandan, D. Ramyachitra

**Affiliations:** Department of Computer Science, Bharathiar University, Coimbatore, 641046 Tamilnadu India

## Abstract

This research work focus on the multiple sequence alignment, as developing an exact multiple sequence alignment for different protein sequences is a difficult computational task. In this research, a hybrid algorithm named Bacterial Foraging Optimization-Genetic Algorithm (BFO-GA) algorithm is aimed to improve the multi-objectives and carrying out measures of multiple sequence alignment. The proposed algorithm employs multi-objectives such as variable gap penalty minimization, maximization of similarity and non-gap percentage. The proposed BFO-GA algorithm is measured with various MSA methods such as T-Coffee, Clustal Omega, Muscle, K-Align, MAFFT, GA, ACO, ABC and PSO. The experiments were taken on four benchmark datasets such as BAliBASE 3.0, Prefab 4.0, SABmark 1.65 and Oxbench 1.3 databases and the outcomes prove that the proposed BFO-GA algorithm obtains better statistical significance results as compared with the other well-known methods. This research study also evaluates the practicability of the alignments of BFO-GA by applying the optimal sequence to predict the phylogenetic tree by using ClustalW2 Phylogeny tool and compare with the existing algorithms by using the Robinson-Foulds (RF) distance performance metric. Lastly, the statistical implication of the proposed algorithm is computed by using the Wilcoxon Matched-Pair Signed- Rank test and also it infers better results.

## Introduction

In Bioinformatics, the sequence alignments are used to show evolutionary relationships by constructing phylogenetic trees. Sequence alignment and phylogenetic analysis are strongly related due to measuring the relatedness of homologous sequence. Generally the protein sequence consists of amino acids, which are linked with each other. Sequence alignment describes the mode of arrangement of protein sequence, in order to distinguish the areas of similarity among them^1^. Aligning refers to matching as many characters as possible from each sequence. Primarily, the sequence alignment is applied to infer functional, morphological and evolutionary relationship between the protein sequences. The alignment of the sequence is used to find similarity level between the query sequence and different database sequences.

Today, there are several sequence alignment techniques are available and this research study concentrates on the multiple sequence alignment. One of the fundamental problems in computational biology is the alignment of multiple sequences of DNA/Protein. The computational approaches which are used to align the Protein/DNA sequences generally falls into two categories: global and local alignments^2^. The multiple sequence alignment comes under the category of global alignment and it’s an adjacent of pairwise alignment to incorporate more than two sequences at a time. Various methods have been implemented on MSA, but these approaches add up under three major classes such as: dynamic programming, Progressive and Iterative methods. In this research work the proposed BFO-GA algorithm comes under the category of Iterative – Progressive method for incorporating the advantage of those methods. The remaining part of this research study is developed as follows: Section 2 illustrates the background field of several methods of solving multiple sequence alignment, Section 3 describes the methodology of MSA multi-objectives and optimization, Section 4 illuminates the proposed algorithm, Section 5 emphasizes the experimental outcomes for the benchmark databases and finally Section 6 spotlights the conclusion and turns over the range for further enhancement.

## Background Study

The dynamic programming is the basic approach to solve multiple sequence alignment problems. Needleman-Wunsch algorithm is the foremost applications of dynamic programming, and it is applied to compare biological sequences^3^. In theoretical, the dynamic programming is applicable to any number of sequences, simply it is computationally expensive in both memory and time. Later than a heuristic search known as progressive technique which is likewise identified as hierarchical or tree method is deployed for multiple sequence alignment^4^. Progressive alignment works by combining pairwise alignments beginning with the highest similar pair and progressing to the most distantly related. Efficient, and the resulting alignments may be reasonable are some of the advantages of the progressive alignment technique. Some of the tools which are developed by using the progressive technique are T-Coffee^5^ and different versions of Clustal. R-Coffee is a web server, which creates highly accurate multiple alignments of non-coding RNA sequences and it is founded on the principle of T-Coffee^6^. The major disadvantage of the progressive technique is the choice of selecting the “most related” sequences.

To overcome the drawbacks of the progressive technique a new approach called Iterative approach, was developed by Gotoh. The iterative approach^7^, focus on improving the accuracy of the initial pairwise alignments, which is the drawback of progressive technique. Now the developments of multiple sequence alignment methods have shifted to iterative algorithms with the progressive approach, such as ProbCons^8^, MAFFT^9^ and MUSCLE^10^. The iterative approach optimizes a scoring function that leads to exact biological alignment. Some more methods that employ progressive and iterative approaches are ClustalW^11^, Clustal Omega^12^, DIALIGN^13^, Match-Box^14^, M-Coffee^15^. COBALT tool incorporates constrains based methods into progressive method^16^. A few tools that based on consistency approaches are K-Align^17^ and Probalign^18^.

The iterative approach is too applied with the stochastic approach and examples of these advances is a Genetic Algorithms^19^, Simulated Annealing^20^, Gibbs Sampling^21^ and Hidden Markov Model^22^. Recently the combinations of iterative and stochastic methods are employed. The genetic algorithm is largely applied for multiple sequence alignment by applying the genetic operators. The approaches that are based on genetic algorithm are MSA-GA^23^, VDGA^24^, GAPAM^23^, RBT-GA^25^ and SAGA^26^. Evolutionary algorithms are also applied as a component for solving the multiple sequence alignment problems. The methods which illustrate the evolutionary algorithms are Particle Swarm Optimization (PSO)^27^, Ant colony optimization (ACO)^28^, Artificial Bee Colony (ABC)^29, 30^, M-BPSO^31^, FTLPSO^32^ and Genetic algorithm with Ant Colony Optimization (GA-ACO)^33^. Most of the MSA methods are prepared utilizing a single objective to align the protein/DNA sequences. In recent times the methods which are developed for multiple sequence alignment problems are based on multi-objectives. The methods which are based on multi-objectives are NAGA II^34^, MSAGMOGA^35^ and MOMSA^36^.

The Sum of Pairs (SP) and the Total Column Score (TCS) are used as performance measures to analyze the algorithms of multiple sequence alignment. Even though several algorithms have been trained for resolving the problem of multiple sequence alignment, but they do not promise to provide the global optimal solution^37, 38^. Hence the BFO-GA algorithm has been suggested for resolving the problem of MSA. And also in this research work, the combination of Similarity, Gap penalty and Non-Gap percentage is employed as the multi-objectives to obtain non-dominated optimal alignment by using the existing and the proposed BFO-GA algorithm.

## Methodology

Normally, the multiple sequence alignments are performed from the primary sequence of a protein^39^. Three or more primary sequences are used to perform the multiple sequence alignment. For a given family M = (m_1_, m_2_, ……. m_n_) of n sequences of fluctuating length L_1_ to L_n_, the finite alphabet ∑ as,

1 $${{\rm{M}}}_{{\rm{i}}}={{\rm{S}}}_{{\rm{i}}1,}{{\rm{S}}}_{{\rm{i}}2,\ldots \ldots \ldots }{{\rm{S}}}_{{\rm{i}}{\rm{L}}{\rm{i}}}(1\le {\rm{i}}\le {\rm{n}}),{{\rm{M}}}_{{\rm{i}}{\rm{j}}}\phantom{\rule{0ex}{0ex}}\in \sum (1\le {\rm{j}}\le {{\rm{L}}}_{{\rm{i}}})$$

where,

∑ consists of 4 characters {A, T, G, C} of nucleotides for DNA Sequences.

∑ consists of 20 characters {A,R,N,D,C,E,Q,G,H,I,L,K,M,F,P,S,T,W,Y,V} of amino acids for protein sequences^35^.

The major performance measure used for multiple sequence alignment is the Sum of Pairs (SP) and Total Column (TC) score. From the matched residues of Protein/DNA, the SP is calculated and the gap penalties are determined by mismatched residues or occurrences of gaps, whereas the similarity is assessed by the substitution matrix score. The similarity matrix score is constructed as 20 × 20 for protein sequences and 4 × 4 for DNA sequences, which represent entire possible transitions between the Protein/DNA sequences. There are two common substitution matrix are available such as Percent Accepted Mutation (PAM) and BLOcks Substitution Matrix (BLOSUM). There are different versions of substitution matrix such as BLOSUM 30, BLOSUM 45, BLOSUM 62, BLOSUM 80, PAM100 and PAM200. In this study, the similarity value is different from the substitution matrix which gives an arithmetical score for matches and mismatches of residues^35^.

Multiple sequence alignment is a complicated problem which consists of three distinct difficulties such as, choice of the sequences, choice of an objective function and optimization of a function. In the proposed BFO-GA algorithm the choice of the sequences is chosen based on the non-dominated optimal solution by using the crowding distance measure. And the optimization of the function is attained by using the BFO-GA algorithm.

### Multi-Objectives and Optimization

In this research work a multi-objective hybrid algorithm named Bacterial Foraging Optimization –Genetic Algorithm is proposed for multiple sequence alignment problems. Typically, the Sum of Pairs (SP) and the Total Column Score (TCS) performance measures are used to find the optimal solution for the MSA Problem. This research study concentrates on three objective functions to determine the optimal solution such as Maximization of Similarity, Minimization of Variable Gap Penalty and Maximization Non-Gap Percentage.

#### Similarity

The computation of position weight matrix for the alignment is generated from the resulted alignment solution. The dominance value (ce) of the leading amino acid or nucleotide in each column is set up as follows:

2 $${\rm{ce}}({\rm{y}})=ma{x}_{x}\{f(x,y)\},\,y=1,2,3\ldots h$$

where f(x, y) is the score value of amino acid or nucleotide x on the column y in the position weight matrix despite of the survival of gaps. h is the sequence alignment length and ce(y) is the dominance value of the dominant amino acid or nucleotide on column y.

The similarity of the alignment SM is defined as the average of dominant value of all columns in the position weight matrix and it is expressed in Eq. 3.

3 $${\rm{Similarity}}\,({\rm{SM}})=\frac{{{\sum }^{}}_{y=1\,}^{h}ce(y)}{h}$$

The candidate alignment SM, which has the greatest probability is identified as the best alignment, if the value of similarity is nearer to 1. The computation of similarity among all sequences is calculated for an alignment.

#### Gap penalty

A gap is an artificial insertions and deletions (indel) into sequence to move similar segments of aligning residues into good alignment. A gap in same columns is not taken into account which has no substance. Different types of gap penalty scores are available such as Constant, Linear, Convex, Affine and profile based variable gap penalties. In this research work affine and variable gap penalty scoring is calculated for the existing and proposed algorithm such as the Genetic Algorithm, Ant Colony Optimization, Artificial Bee Colony, Particle Swarm Optimization and BFO-GA algorithm to anticipate better outcomes.

#### Affine gap penalty

Insertions and deletions are scored using an affine gap penalty that penalizes the gap once for opening and then proportionally to its length dependent. Two parameters are applied, namely gap opening and gap extension^40^. The formula for calculating the affine gap penalty in the pairwise alignment of rows x and y is determined by

4 $$Ga{p}_{xy}({\rm{c}})=Ga{p}_{open}+Ga{p}_{extend}\,(g-1),\,where\,g > 1$$

Gap_open_ → cost of opening a gap

Gap_extend_ → cost of extending a gap by one more space

g → length of gap string

The optimization of affine gap is to group the gaps together, which will minimize the affine gap penalty.

#### Variable gap penalty

The general usage of affine gap penalty is not appropriate for multiple sequence alignment. The gap penalty values of affine are constant and applied equally in all positions, thus the value of gap penalty is determinant. A new position-specific gap penalty is used where the gap values vary according to the residues to find the optimal alignment. MAFFT and ClustalW tools adopted this type of gap penalty.

The initial gap penalties are calculated based on the fixed values set by users. Mainly, there are two gap penalties are applied.

**Gap Opening Penalty (GOP):** - indicates the cost of opening a new gap of any length.

**Gap Extension Penalty (GEP)**: - indicates the cost of every item in a gap.

Afterwards, the local gap opening and extension penalties are changed according to the following factors^11^: The gap opening penalties are recalculated based on the factors such as dependence on the weight matrix (Off-diagonal values of the matrix), depends on the similarity of the sequences (Percent identity of two sequences) and depending on the lengths of the sequences.

5 $${{\rm{Gap}}}_{{\rm{open}}}\to \{{{\rm{Gap}}}_{{\rm{open}}}+\,\mathrm{log}[{\rm{\min }}({\rm{R}},{\rm{T}})]\}\ast ({\rm{n}})\ast ({\rm{m}})$$

where,

R and T are a length of 2 sequences,

n- Average of residue mismatch score,

m- Percent identity scaling factor

The gap extension penalties are recalculated based on the following elements.

- Depending on the difference in the lengths of the sequences.
  6 $${\rm{Gapext}}\to {\rm{Gapext}}\ast [1.0+|\,\mathrm{log}({\rm{R}}/{\rm{T}})|]$$
  where, R and T are the lengths of the two sequences.
- Position-specific gap penalties (*counting the frequency of each residue at either end of gaps in alignments, store in table*)
*GOT- gap opening penalty table which traces the penalty along the length of sequences i, for each pair of sequences i and j*.
*GET- gap extension penalty table*
- Lowered gap penalties at existing gaps.
  7 $${\rm{GOT}}\to {\rm{GOT}}\ast 0.3\ast ({\rm{number}}\,{\rm{of}}\,{\rm{sequences}}\,{\rm{without}}\,{\rm{a}}\,{\rm{gap}}/{\rm{number}}\,{\rm{of}}\,{\rm{sequences}})$$
- Increased gap penalties near existing gaps.
  8 $${\rm{GOT}}\to {\rm{GOT}}\ast \{2+[(8-\mathrm{distance}\,{\rm{from}}\,{\rm{gap}})\ast 2]/8\}$$
- Reduced gap penalties in hydrophilic stretches
  9 $${\rm{GOT}}\to {\rm{GOT}}\ast 0.5\,({\rm{if}}\,{\rm{there}}\,{\rm{is}}\,{\rm{hydrophilic}}\,{\rm{residue}}\,{\rm{at}}\,{\rm{xth}}\,{\rm{position}})$$
- Residue-specific penalties (*no hydrophilic stretch and gap, GOP is multiplied by one of the 20 numbers*).

10 $${\rm{GOT}}={\rm{GOT}}\ast T[{S}_{x}]$$

where S_x_ is the value of residue located on the x^th^ position of sequence S in the residue table.

Finally the GOP and GEP are calculated based on equations- 7, 8, 9 and 10.

11 $${\rm{GOP}}({\rm{n}},{\rm{m}})={\rm{GOT}}({\rm{n}})+{\rm{GOT}}({\rm{m}})$$

12 $${\rm{GEP}}({\rm{n}},{\rm{m}})={\rm{GET}}({\rm{m}})$$

Based on these factors the variable gap penalty is inserted into the input of Protein/DNA sequence.

#### Non gap percentage

The arithmetic significance of an alignment score usually depends on a theoretical form of non-gapped alignments. Some methodologies generally use too much of gaps to raise the identities in alignment. The non-gap percentage is defined as the total number of amino acids in the sequences with respect to the number of gaps in the sequences^41^.

13 $${\rm{NGP}}=\frac{Total\,number\,of\,gaps\,in\,the\,sequences}{Total\,number\,of\,amino\,acids\,in\,the\,sequences}\times 100$$

### Non-dominated Optimal Solution

Generally, the objectives of the optimization problem differ from each other. If one of the objectives achieves the optimal solution by maximizing the value while the other objective function achieves an optimal solution but if the value gets minimized which needs a concession for the final result. The domination plays a major role in multi-objective optimization, where the solution d_m_ is assumed to dominate another solution d_n_ if the subsequent two conditions are true:

- The solution of d_m_ is not poorer than d_n_ in all objective functions.
- The solution d_m_ is definitely superior to d_n_ at least in one objective function.

This contributes to the characterization of Pareto-optimal solution^37^. The complexity for the non-dominated sorting based multi-objective evolutionary is $\,O{(MN)}^{2}$, where M is the number of objective functions and N is the total number of people in the population. Once applied the non-dominated sorting algorithm, the diversity among non-dominated individuals are introduced using crowding distance and the selection is pulled in by employing the crowded tournament selection. This approach is able to discover much better spread of solutions and enhanced convergence close to the true Pareto-Optimal front solution^42^.

## Proposed BFO-GA Algorithm

**Figure 1:**
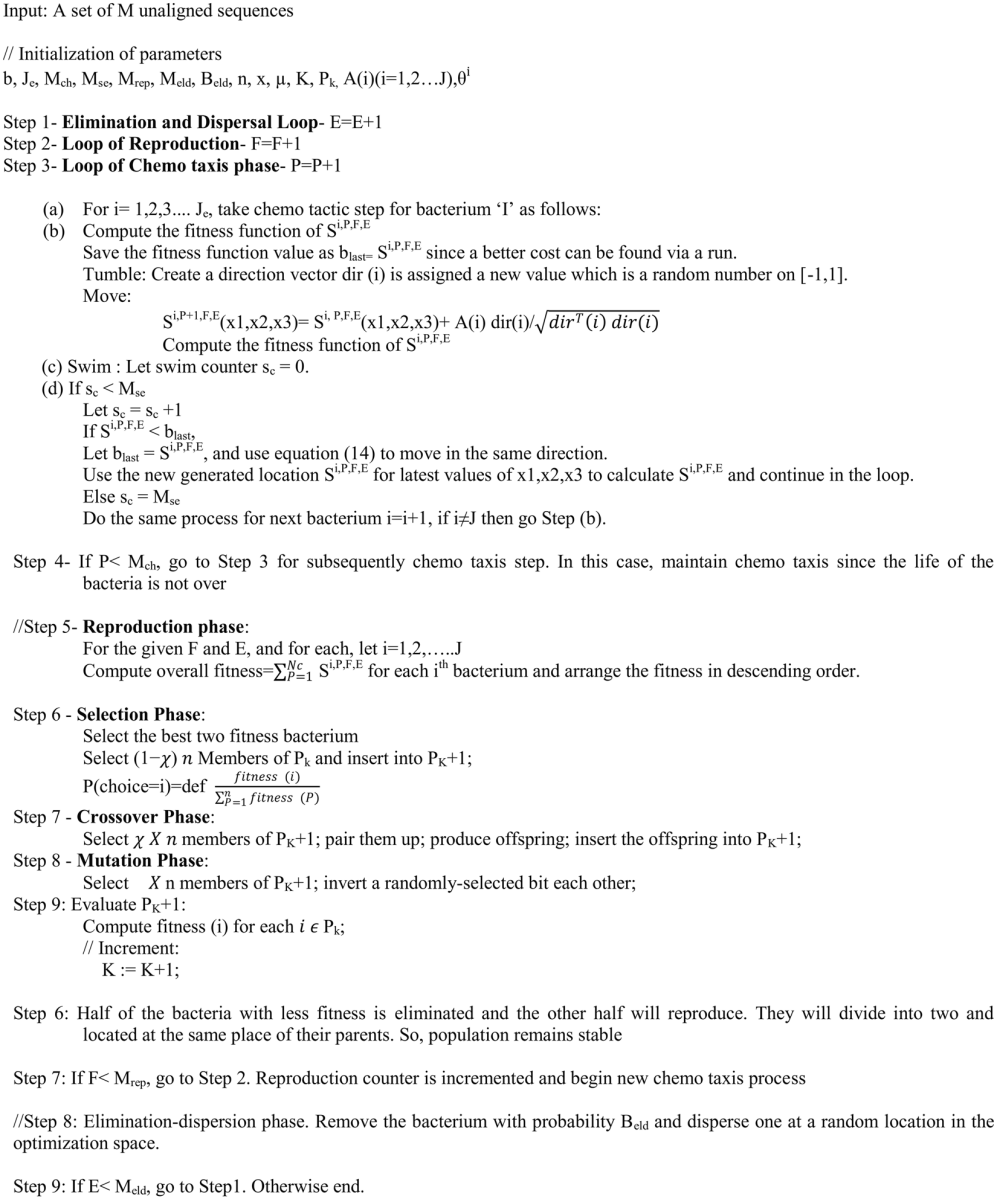
Pseudo code of the proposed BFO-GA algorithm.

The non-dominated optimal solution for the multiple sequence alignment problems is predicted by using the proposed BFO-GA algorithm. The proposed BFO-GA algorithm is a scattered optimization process, which is founded on the individual and group behavior of *E. coli* bacteria. It consists of chemotaxis, swarming, reproduction phase, selection, crossover, mutation, elimination and dispersal phase. The chemotaxis is a central step in BFO-GA algorithm, where a bacterium takes steps over the foraging site in order to gain the alignment with higher fitness value. All of the above phases for the BFO-GA algorithm are iterated until the maximum cycle is reached. The pseudo code for the proposed algorithm is given in Fig. 1.

The parameters which are employed in the Pseudo code of the proposed BFO-GA algorithm in Fig. 1 are as follows,

*b* - *Dimension of search space. It is a quantity of parameters to be optimized*.

J_e_ *- Total Number of bacteria in the population*

M_ch_ *- The number of chemotactic steps*

M_se_ *- The number of swim lengths*

M_rep_ *- The number of reproduction steps*

M_eld_ *- The number of elimination and dispersal steps*

B_eld_ *- Probability of elimination and dispersal*

n*- Number of Individuals in a population*

*χ* - *fraction of the population to be replaced by cross over*

*μ* – *mutation rate*

*Initialize generation* = *0*;

K=0;

P_k_ = *a population of n randomly-generated individuals;*

**Figure 2:**
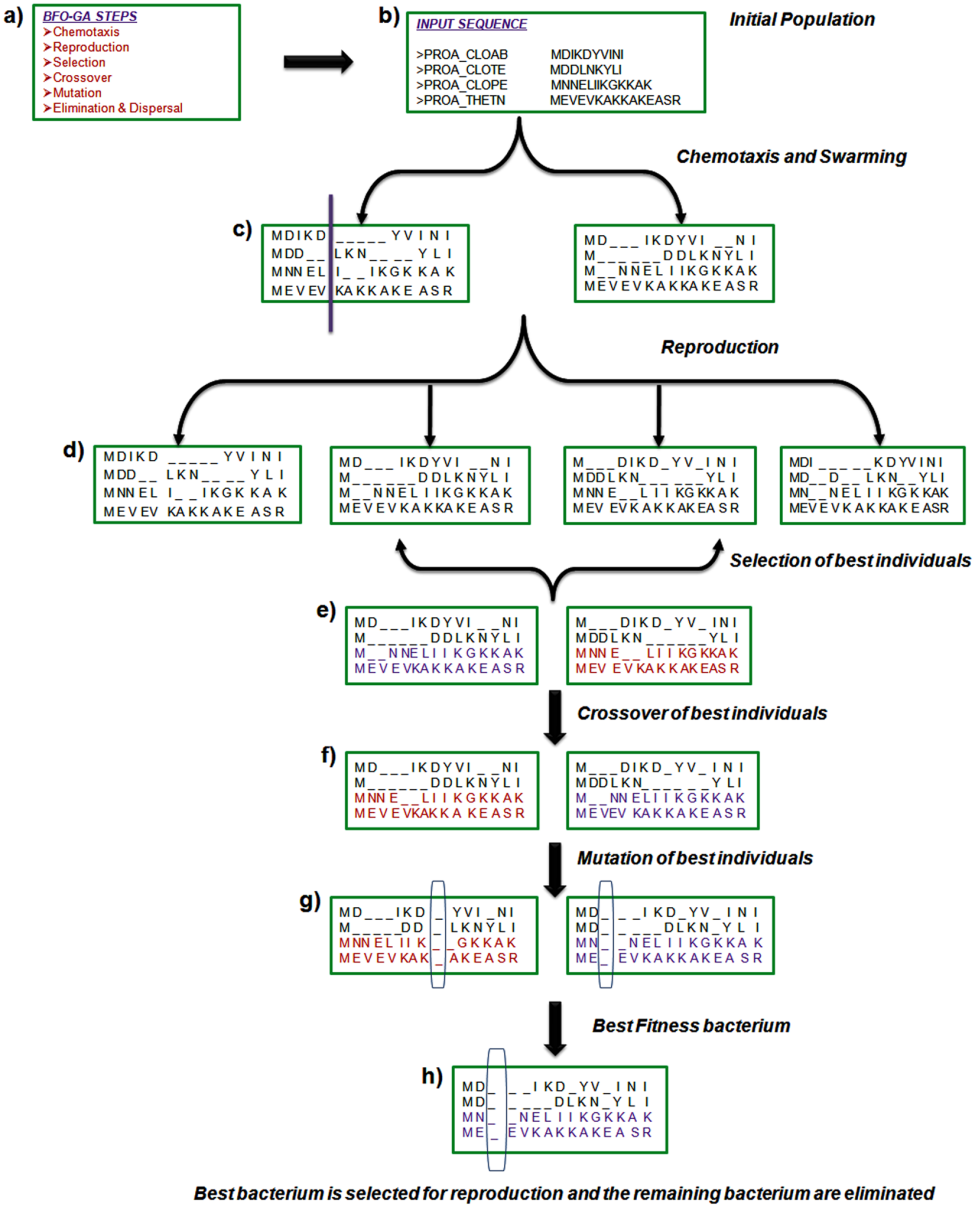
Flowchart of the proposed BFO-GA algorithm.

The overall framework for the proposed BFO-GA algorithm is shown in Fig. 2. Figure 2(a) shows the major steps involved in the proposed BFO-GA algorithm.

### Initialization of bacterium in employing phase

The set of unaligned Protein/DNA sequences is presented as an input. The input sequences are in different length. In order to align the sequences, they should be in same length. The gaps are inserted randomly to shuffle the residues in between them to get the optimal alignment. The percentage of gaps added to the largest sequence should be less than 20% of the longest sequence length^35^. After this the other sequences, adjust to the largest sequence length to get the same length of all sequences. The evaluation of the population using employed bacterium for calculating new food sources is completed. The Fig. 2(b) shows the initial population for employing the BFO-GA algorithm.

### Chemotaxis

The Swimming and tumbling characteristics of bacteria is used to search for the food and it is known as chemotaxis. If a bacterium is said to be ‘swimming’, it impresses in a pre-defined direction. If it is supposed to be ‘tumbling’, it impresses in an entirely different way. Then movement of i^th^ bacterium in P^th^ chemotaxis step can be represented by following equation.

14 $${{\rm{S}}}^{{\rm{i}},{\rm{P}}+1,{\rm{F}},{\rm{E}}}({\rm{x}}1,{\rm{x}}2,{\rm{x}}3)={{\rm{S}}}^{{\rm{i}},{\rm{P}},{\rm{F}},{\rm{E}}}({\rm{x}}1,{\rm{x}}2,{\rm{x}}3)+{\rm{A}}({\rm{i}}){\rm{dir}}({\rm{i}})/\sqrt{di{r}^{T}(i)dir(i)}$$

where,

dir(i) → direction vector. dir (i) is a random number lying between [−1, 1].

S^i,P+1,F,E^(x1, x2, x3) → position of ith bacterium at a point in x1, x2, x3 coordinate system, in P^th^ Chemotaxis, F^th^ reproduction and E^th^ elimination and dispersal step.

C(i) → unit run-length of a bacterium

In this proposed BFO-GA algorithm, the swimming length of the bacteria in multiple sequence alignment is randomly applied by the user. Only, in this research work the proposed algorithm gives better outcomes when the bacterium makes a motion in a forward direction with a swimming length of 5.

### Swarming

In favor of the bacteria to pass at the highest food location, it is trusted that the optimum bacterium till a point of time in the search time should make an endeavor to draw in other bacteria so that together they unite at the desired location more quickly. To accomplish this, a penalty function based upon the degraded non-dominated sorting algorithm is executed to determine the fittest bacterium which has higher crowding distance and lower social status. The relative lengths of each bacterium from the fittest bacterium till that search duration are added to the original cost function. Figure 2(c) illustrates the chemotaxis and swarming length of 5 with the forward direction for the initial population.

### Reproduction

The singular set of bacteria, after getting changed through several chemotactic stages reaches the breeding phase. At this stage, the best set of bacteria gets divided into two groups. The healthier half replaces with the other half of bacteria, which gets eliminated, due to their poorer foraging abilities. This formulates the population of bacteria constant in the development process. The reproduction of the initial population for the protein sequences is shown in Fig. 2(d).

### Selection Phase

In selection phase, the sorting of individuals is done in the mating pool according to their fitness and then every two best individuals are selected for crossover. The best fitness bacterium is calculated by scoring each alignment according to the Multi-objectives (Equations 2–13). The fast non-dominated sorting algorithm is executed to relieve the best bacterium which has higher crowding distance and lower rank^42^. The choice of the best bacterium is done by crowded tournament selection. Based on the fitness value, every two best individuals are selected for crossover and it is shown in Fig. 2(e).

### Crossover Phase

The single point crossover is applied to generate new offsprings from the parents. Again the fitness is calculated and the best bacterium is selected. For every two best individuals, the initialization of parameter value for performing the crossover operation in BFO-GA is set to 0.3 and it is shown in Fig. 2(f).

### Mutation Phase

With the final best bacterium the mutation operation is done to generate new offsprings which perform modifications to provide the possible difference for the offspring alignments. It avoids the premature convergence of alignment. Now the fitness value is calculated and the best bacterium is identified. For every two best individuals, the initialization of parameter value for performing the mutation in BFO-GA algorithm, the parameter is set to 0.8 and it is shown in Fig. 2(g).

### Elimination and dispersal

In the evolutionary process, an unexpected event can take place, which may drastically alter the process of evolution and cause the elimination of the set of bacteria and disperse them to a novel environment. As an alternative of raising up the usual chemotactic growth of the set of bacteria, this unknown event may pose a raw set of bacteria nearer to the food location. From a broader perspective, elimination and dispersal are part of the population level long distance motile behavior. In optimization, it aids in thinning out the behavior of stagnation which normally takes place in parallel search algorithms. The worst bacterium is replaced by the best developed offspring if their fitness values are better than worst bacterium. The best bacterium is selected for reproduction (Fig. 2(h)), and the remaining bacterium are eliminated.

## Experimental Results

In this research study, the proposed algorithm is examined with the well-known benchmark datasets for analyzing the execution of the algorithm based on the potency. In summation, the public presentation of the proposed algorithm has been assessed by comparing with several optimization techniques, namely Genetic Algorithm (GA), Ant Colony Optimization (ACO), Artificial Bee Colony (ABC), Particle Swarm Optimization (PSO) and existing online tools namely T-Coffee, Muscle, K-Align, MAFFT and Clustal Omega.

### Performance Measures

This research focuses on the performance measures such as the ratio of pairs correctly aligned namely Sum of Pairs (SP), the ratio of the columns correctly aligned namely Total Column Score (TCS) and the multi-objectives such as maximization of similarity, gap penalty and Non-Gap percentage. The experiments are taken out in 2 X Intel Xenon E5-2670 V2 (2.5 GHz/10-core) CPU with 64 GB of memory, running Cent OS and the proposed BFO-GA algorithm was implemented in Java.

The first performance standard used in this work named Sum-of pairs (SP) and it is set as the number of correctly aligned amino acids or residues with respect to the total number of residue pairs in the reference alignment. Consider the example test alignment of size R*T and a reference alignment of size R*Tr, where X is the number of sequences and T,Tr are the total number of columns in the test and reference alignment. Here $\,{B}_{i1},\,{B}_{i2}\ldots \mathrm{..}{B}_{iX}$, is the i^th^ column in the alignment, ${F}_{iab}=1$ is defined for each pair of residues ${B}_{ia}\,\,and\,{B}_{ib}$ only if ${A}_{ia}and\,{A}_{ib}$ are aligned with each other in the reference alignment, otherwise ${F}_{iab}=0.$ The score $S{P}_{i}$ for the ith column will be the sum of ${F}_{iab}\,$for all pairs of residues in this column is represented in Eq. 15.

15 $${{S{P}_{i}=\sum }^{}}_{a=1,a\ne b}^{X}{{\sum }^{}}_{b=1}^{X}{F}_{iab}$$

Similarly $S{P}_{ri}$ is the score $S{P}_{i}\,$for the i^th^ column in the reference alignment.

The sum-of-pairs score for the test alignment is defined in Eq. 16

16 $${{{\rm{SP}}=\sum }^{}}_{i=1}^{T}S{P}_{i}/{{\sum }^{}}_{i=1}^{Tr}S{P}_{ri}$$

And the second most common scoring scheme for Multiple Sequence Alignment is Total Column Score (TCS). Generally, TCS is defined as the number of correctly aligned columns with respect to the total number of columns in the reference alignment. Consider the example test alignment of size R × T and a reference alignment of size R × Tr, where R is the number of sequences and T, Tr are the total number of columns in the test and reference alignment. Here the score is defined as $Co{l}_{i}$ = 1 if all the residues are aligned in the reference alignment, otherwise $Co{l}_{i}=0.$

The total column score for test alignment is represented in Eq. 17.

17 $$TCS={{\sum }^{}}_{i=1}^{T}Co{l}_{i}/T$$

### Implementation and Discussion

In this work the universally known benchmark datasets such as Benchmark Alignment Database (BAliBASE 3.0)^43^, Prefab 4.0^10^, SABmark 1.65^44^ and Oxbench 1.3^45^ is used to examine and compare with various multiple sequence alignment methods. The BAliBASE 3.0 database contains 6255 protein sequences in total length. It includes five diverse reference sets, namely RV1, RV2, RV3, RV4 and RV5.

And the Sabmark database contains 3280 protein sequences in Twilight Zone families. That is the sequence similarity lies between 0–25% identity and common evolutionary origin cannot be established between most pairs of the sequences. The Prefab benchmark database contains 1682 reference alignments. Finally the Oxbench database consists of reference alignments in the master reference set and 605 sequences in the full reference set. Choosing of gap penalty for the benchmark datasets used in this study are keyed out based on the different gap penalty values such as 2%, 5%, 10%, 15% and 19%. It was found that 19% of gap value among various percentages gave better answers and hence it was specified.

**Figure 3:**
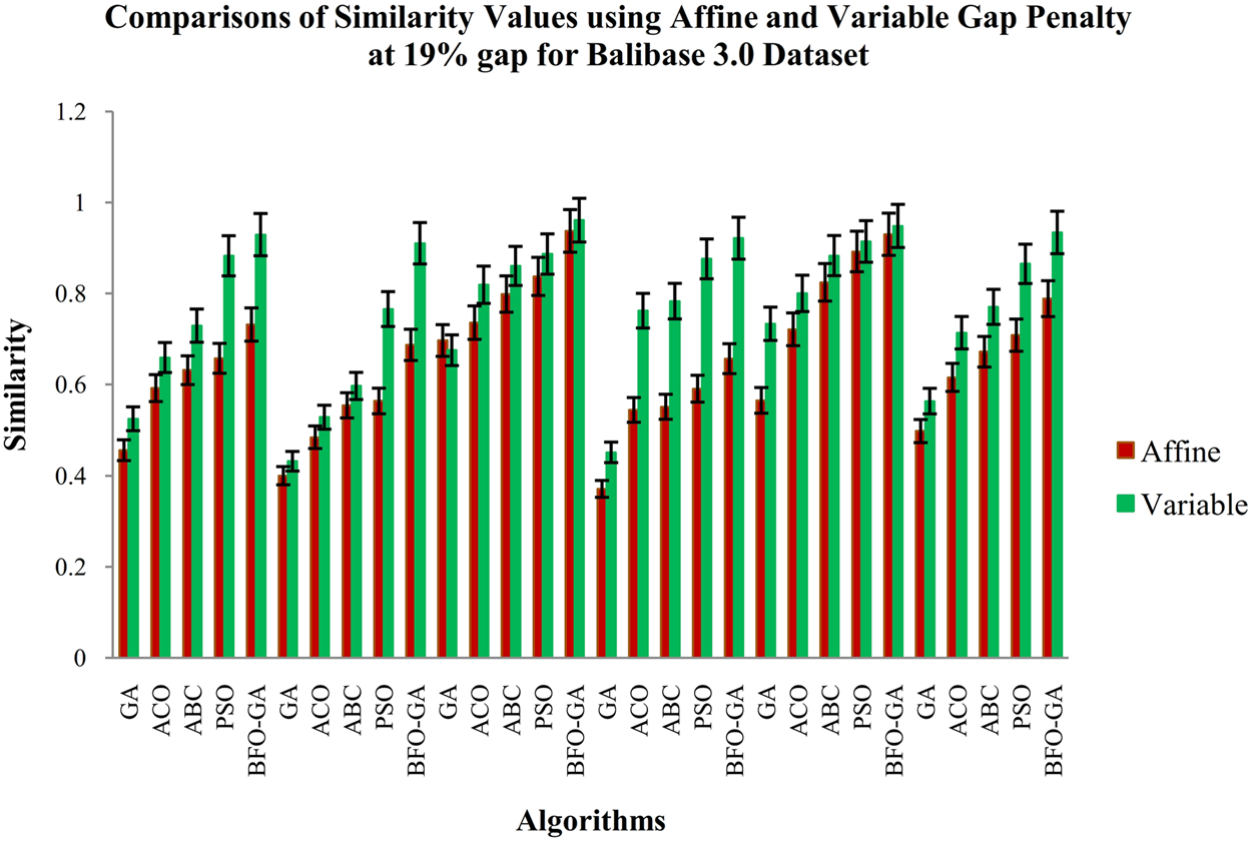
Comparisons of Similarity values for BAliBASE datasets using Affine and Variable Gap penalty for the proposed and existing algorithms.

The Fig. 3 shows the average results for 19% of gap value and 500 numbers of generations. In this study two sets of observational results are acquired, where the first one is to count the values of objective functions such as similarity, gap penalty and non-gap percentage for five algorithms (GA, ACO, ABC, PSO and the proposed BFO-GA algorithm). The second one is to calculate the performance measures, namely SP and TCS values. The proposed algorithm has been performed for 25 runs and the intermediate results are exhibited.

**Figure 4:**
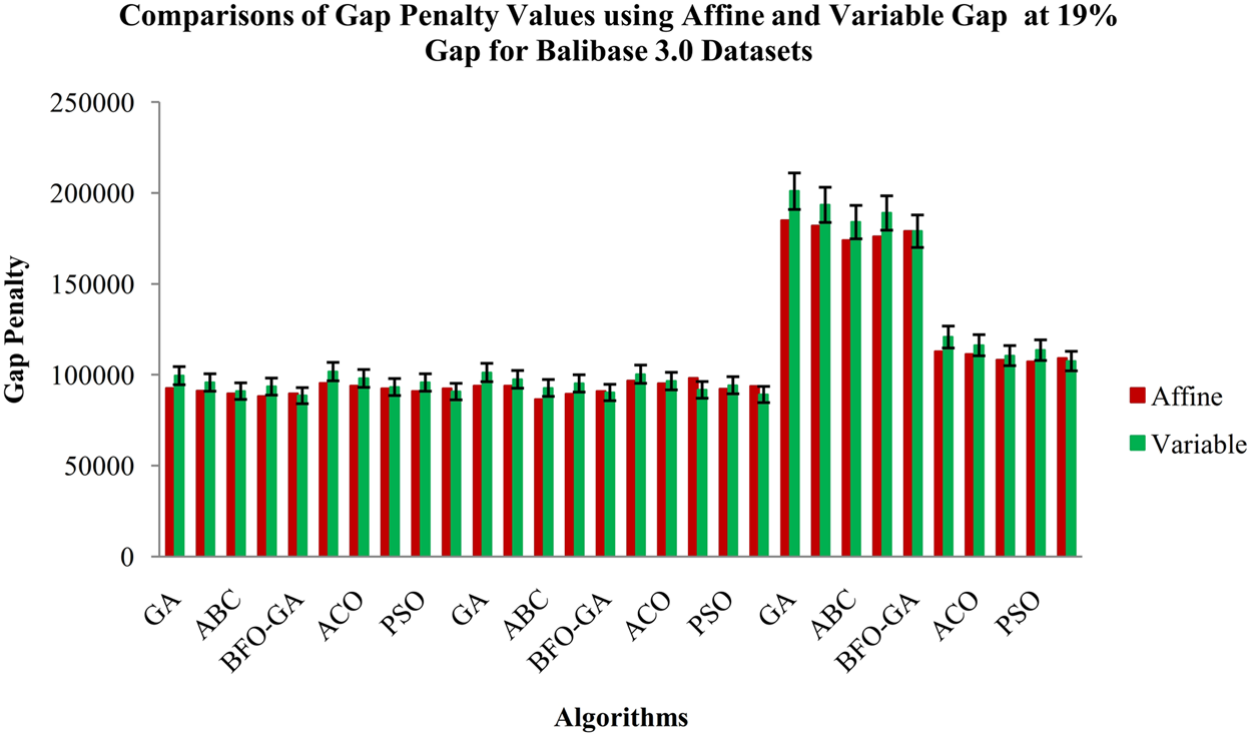
Comparisons of Gap Penalty values for BAliBASE datasets using Affine and Variable Gap penalty for the proposed and existing algorithms.

**Figure 5:**
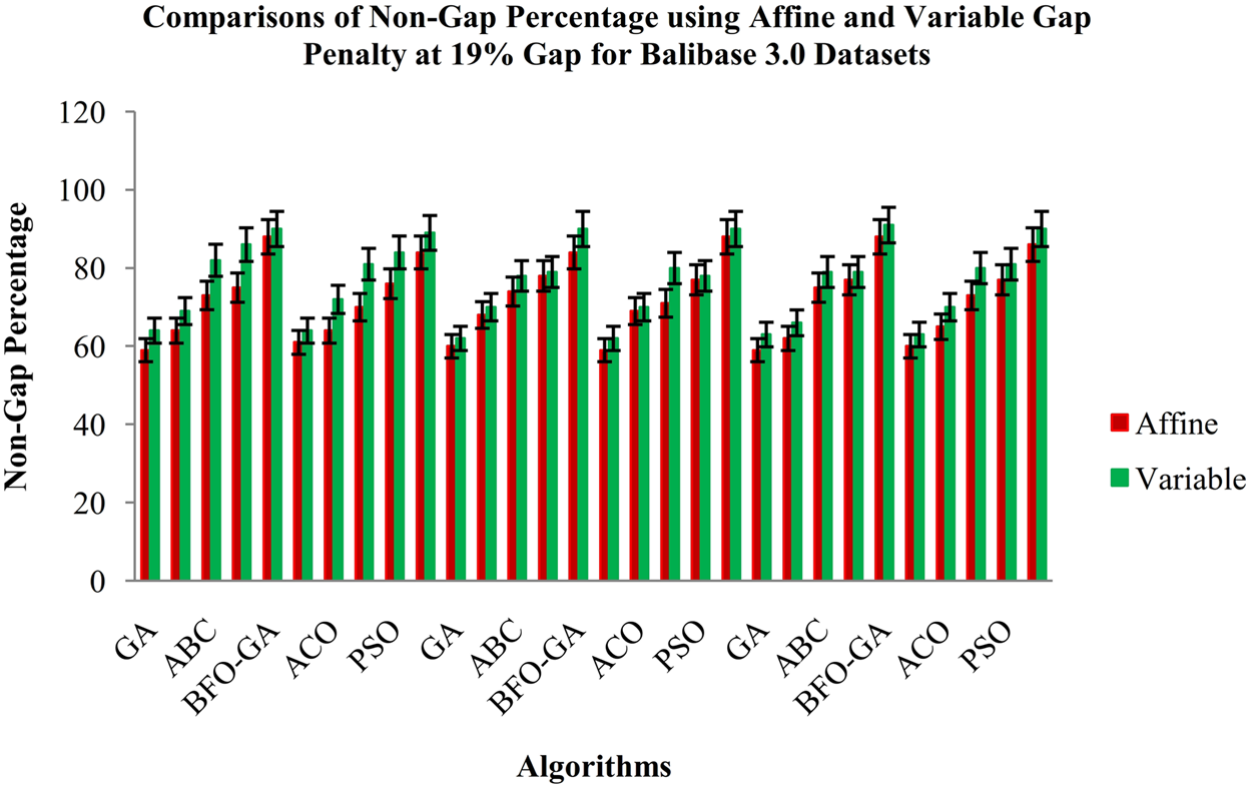
Comparisons of Non-Gap Percentage values for BAliBASE 3.0 datasets using Affine and Variable Gap penalty for the proposed and existing algorithms.

**Figure 6:**
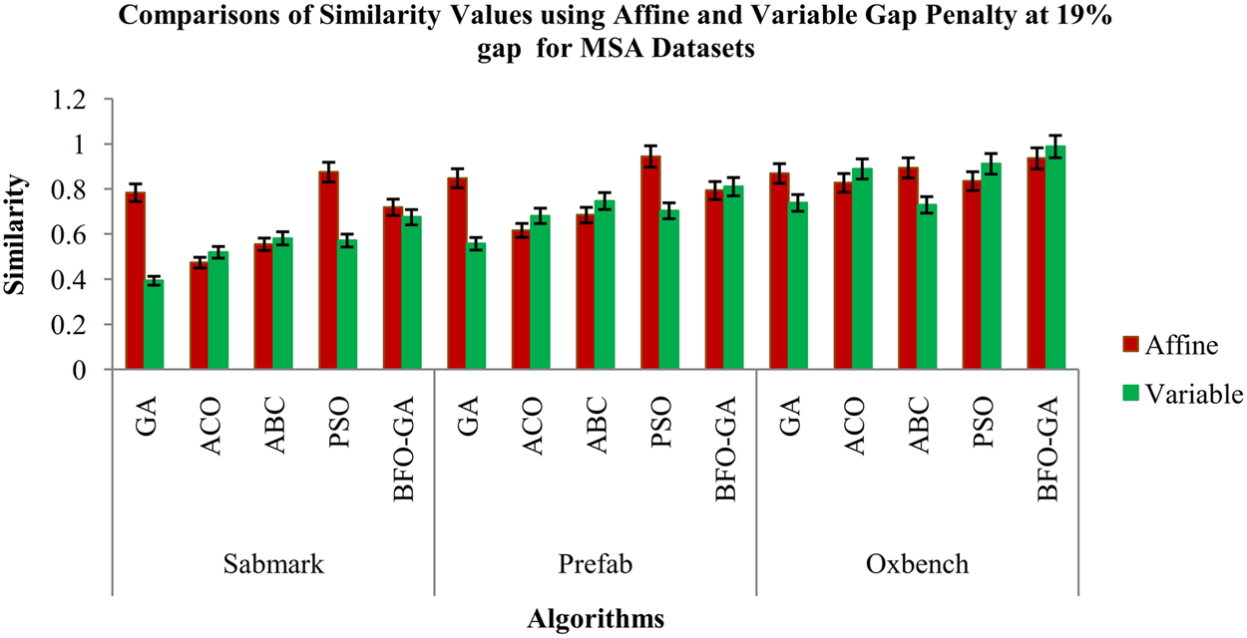
Comparisons of Similarity values for MSA datasets using Affine and Variable Gap penalty for proposed and existing algorithms.

**Figure 7:**
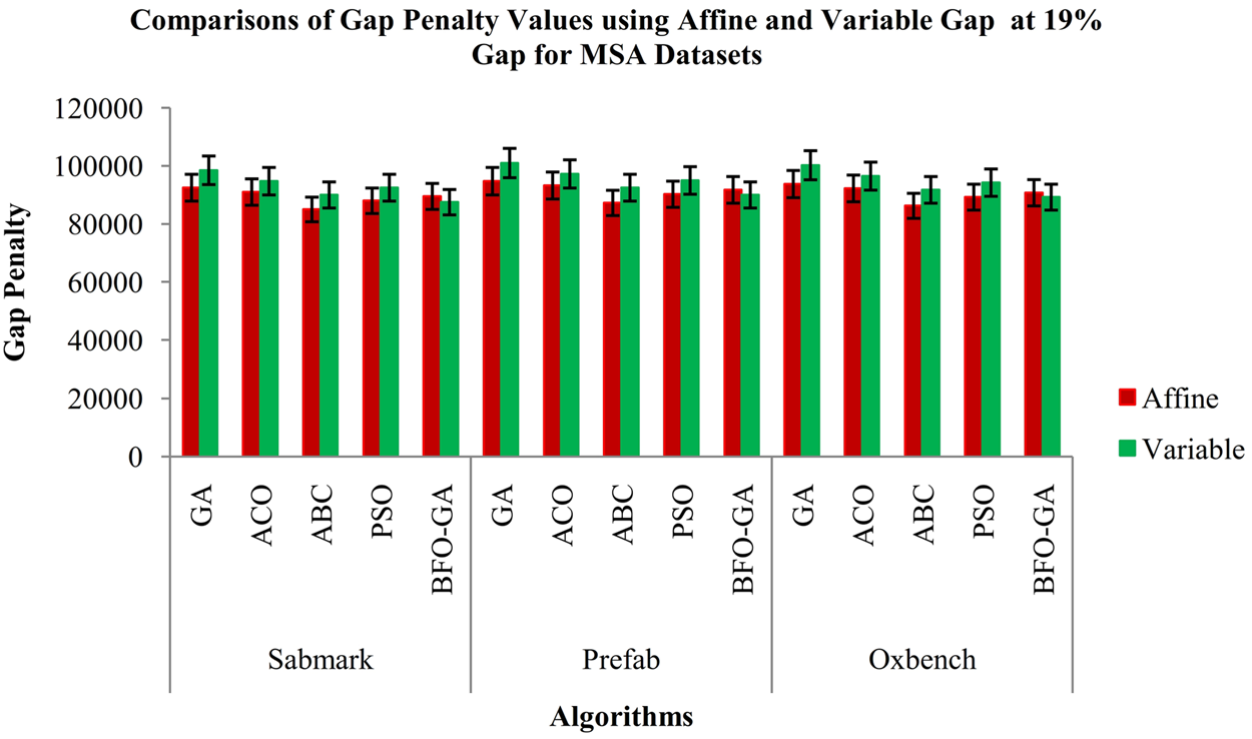
Comparisons of Affine and Variable Gap Penalty values for MSA Datasets using the proposed and existing algorithms.

**Figure 8:**
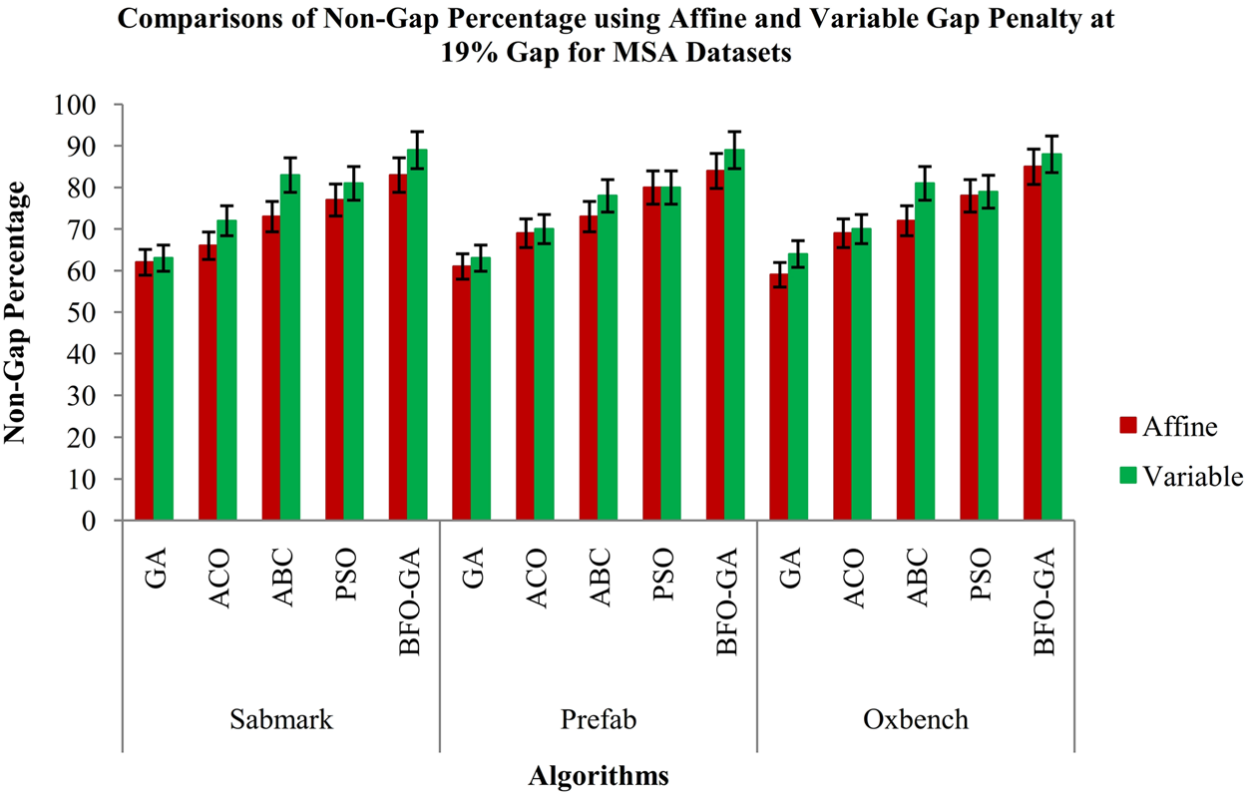
Comparisons of Non-Gap Percentage values for MSA datasets using Affine and Variable Gap penalty for the proposed and existing algorithms.

From the Fig. 3, it is inferred that the proposed BFO-GA algorithm achieves higher performance for all multi-objective values than the existing algorithms. For all the datasets, the proposed algorithm provides more expert results for the value of similarity, gap values and non-gap percentage. It is also found that the similarity and non gap percentage values for variable gap penalty is better than the values achieved by using an affine gap penalty. The comparisons of similarity using affine and variable gap penalty of five reference BAliBASE datasets for proposed and existing algorithms are shown in Fig. 3. The comparisons of Affine and Variable gap penalty of five reference BAliBASE 3.0 datasets for the proposed and existing algorithms is shown in Fig. 4. The comparisons of non- gap percentage for the alignment of five reference BAliBASE 3.0 datasets for the proposed and existing algorithms is shown in Fig. 5. The comparisons of similarity using affine and variable gap penalty of well-known benchmark datasets such as Sabmark, Prefab and Oxbench for proposed and existing algorithms are shown in Fig. 6. Likewise, the comparisons of Affine and Variable gap penalty of alignment benchmark datasets in the above mentioned for the proposed and existing algorithms are shown in Fig. 7. Ultimately, the comparisons of non- gap percentage for the alignment benchmark datasets for the proposed and existing algorithms is shown in Fig. 8.

**Figure 9:**
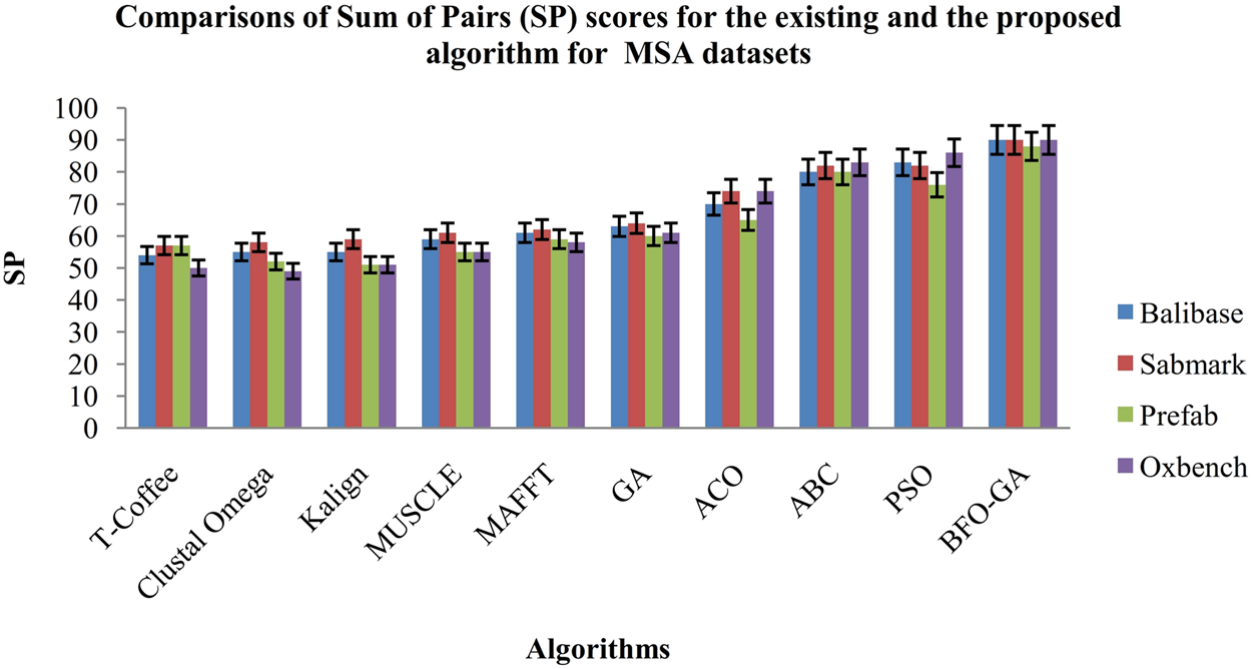
Comparison of Sum of Pairs (SP) scores for the existing and the proposed algorithm for the MSA Datasets.

**Figure 10:**
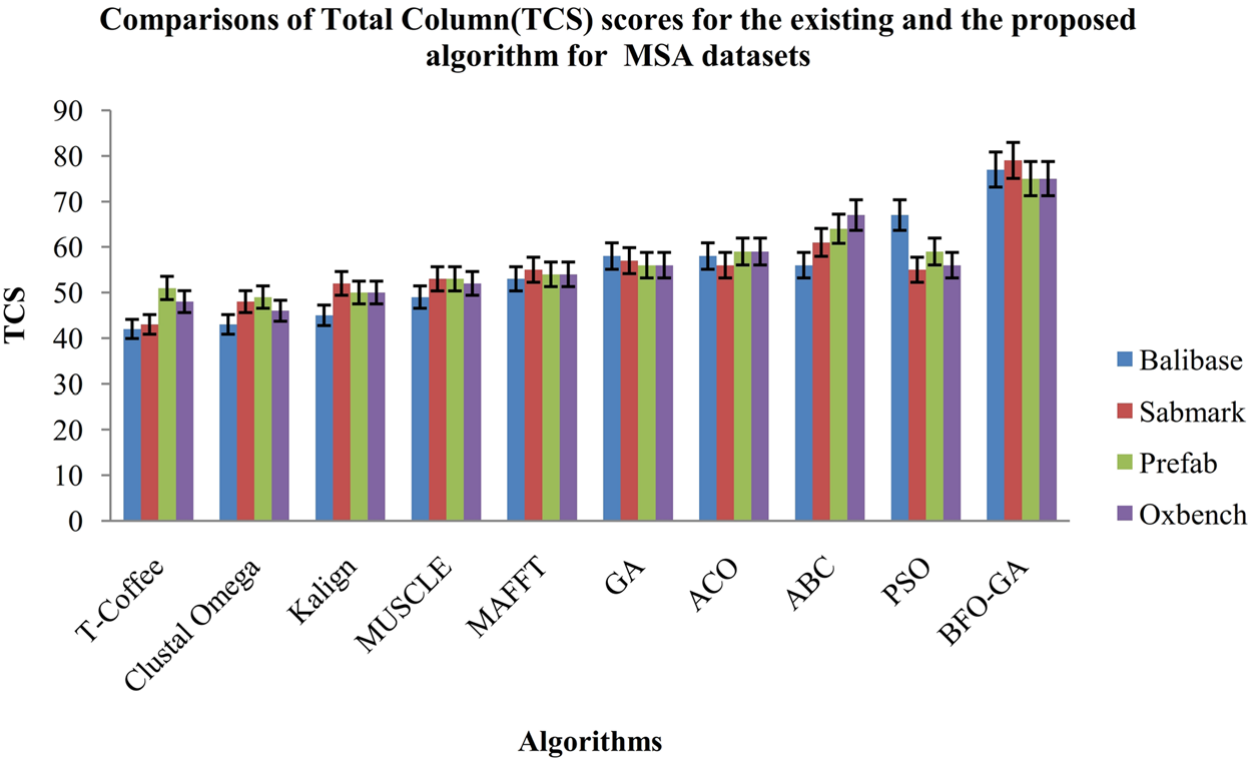
Comparison of Total Column Score (TCS) scores for the existing and the proposed algorithm for the MSA Datasets.

The performance standards such as the Sum-of-Pairs (SP) and Total Column Score (TCS) for the proposed algorithm are compared with existing algorithms (GA, ACO, ABC, PSO) and also with various online MSA tools such as T-Coffee, Muscle, K-Align, MAFFT and Clustal Omega. The Figs 9 and 10 shows the performance results of the SP and TCS values at 19% of gap percentage. From the Figs 9 and 10, it is concluded that the proposed BFO-GA algorithm achieves higher performance outcomes for every dataset for both Sum-Of-Pairs and Total Column Scores. From the observational results, it in inferring that the similarity and non-gap percentage values increases and gap penalty value decreases gradually when increasing the iterations of execution. Also the values of multiple sequence alignment are fully dependant on the input sequence characters. Every performance measures are fluctuated during the first four runs of the experiment and in the later runs, consistency was observed.

**Figure 11:**
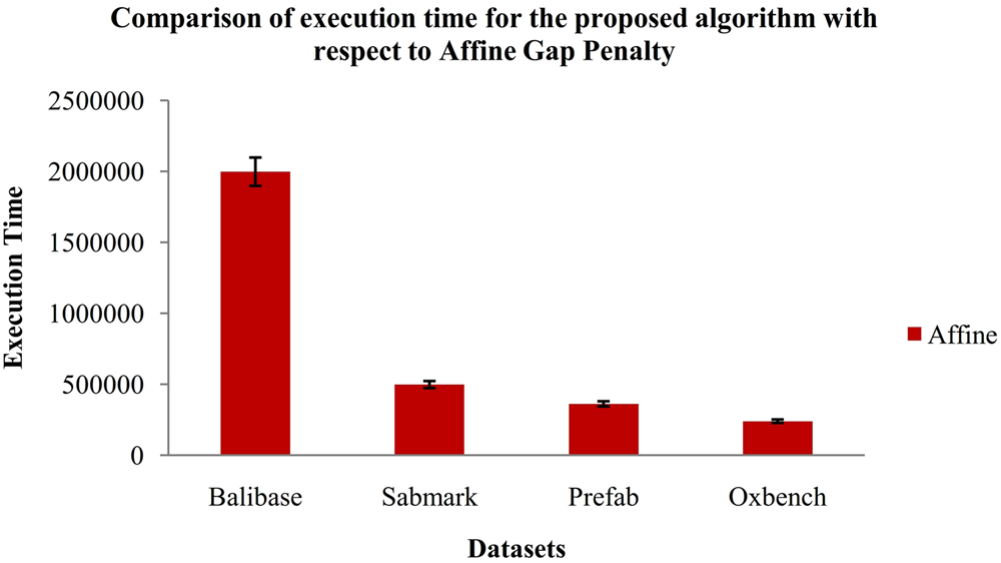
Execution time for the proposed BFO-GA algorithm with respect to Affine Gap Penalty.

**Figure 12:**
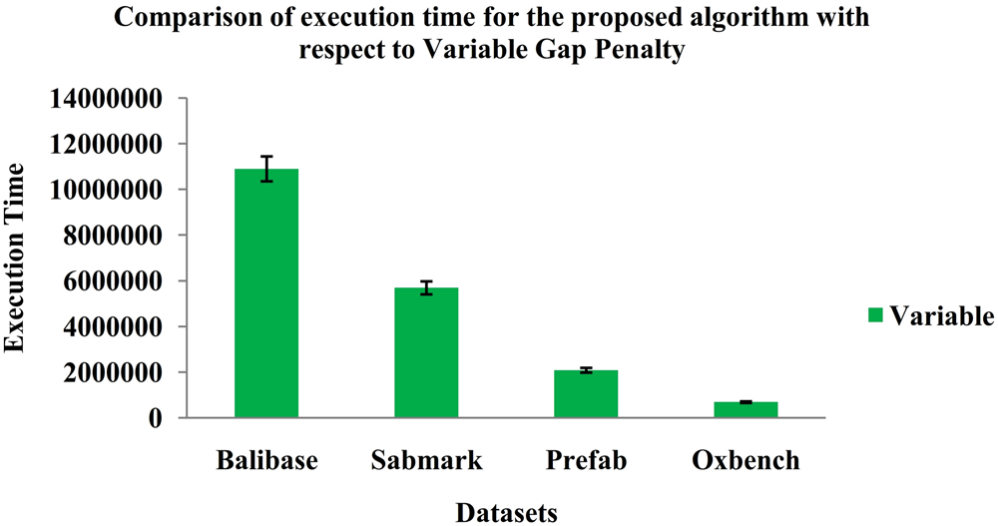
Execution time for the proposed BFO-GA algorithm with respect to Variable Gap Penalty.

The final stage yields the statistical significance of the proposed algorithm which is estimated using non-parametric test, namely Wilcoxon Matched-Pair Signed-Rank test between each pair of methods by using significant confidence level of 5% (P-value < 0.05). Each entry in the Table 1 consists of P-value assigned by Wilcoxon Matched-Pair Signed-Rank test for the divergence between the pair of methods. The upper right corner of the matrix is obtained from SP score and the lower-left corner is obtained from TCS score. The execution time for the proposed BFO-GA algorithm with respect to affine and variable gap penalties is shown in Figs 11 and 12.

**Table 1:** Statistical significance of proposed and existing algorithms for MSA benchmark datasets.

	T-Coffee	Clustal Omega	Kalign	MUSCLE	MAFFT	GA	ACO	ABC	PSO	BFO-GA
T-Coffee		**0.705**	**0.713**	**0.141**	<10^−10^	<10^−10^	<10^−10^	<10^−10^	<10^−10^	<10^−10^
Clustal Omega	**1**		**0.414**	<10^−10^	<10^−10^	<10^−10^	<10^−10^	<10^−10^	<10^−10^	<10^−10^
Kalign	**0.144**	**0.066**		<10^−10^	<10^−10^	**0.068**	<10^−10^	<10^−10^	<10^−10^	<10^−10^
MUSCLE	<10^−10^	<10^−10^	**0.068**		**0.068**	<10^−10^	**0.068**	<10^−10^	<10^−10^	<10^−10^
MAFFT	<10^−10^	<10^−10^	<10^−10^	**0.066**		**0.068**	<10^−10^	<10^−10^	<10^−10^	<10^−10^
GA	**0.068**	<10^−10^	<10^−10^	<10^−10^	<10^−10^		**0.068**	<10^−10^	<10^−10^	<10^−10^
ACO	<10^−10^	<10^−10^	<10^−10^	<10^−10^	<10^−10^	**0.276**		**0.068**	**0.068**	<10^−10^
ABC	<10^−10^	<10^−10^	<10^−10^	<10^−10^	<10^−10^	**0.144**	**0.144**		**1**	<10^−10^
PSO	<10^−10^	<10^−10^	<10^−10^	<10^−10^	<10^−10^	**0.285**	**1**	**0.581**		<10^−10^
**BFO-GA**	<10^−10^	<10^−10^	<10^−10^	<10^−10^	<10^−10^	<10^−10^	<10^−10^	<10^−10^	<10^−10^

### Phylogenetic Tree Construction

In this research four well-known benchmark datasets such as BAliBASE 3.0, Prefab 4.0, SABmark 1.65 and Oxbench 1.3 are used for comparing the proposed BFO-GA algorithm with the other existing algorithms. After performing the MSA, the resulting alignments are passed to the online tool ClustalW2^46^ to reconstruct the phylogenetic trees of the families. The Supplementary Fig. 1 shows the reference phylogeny for a subset of one reference family in BaliBASE 3.0 named RV 3, as well as the consequent phylogenetic trees reconstructed from the alignments obtained from the other four algorithms. Robinson-Foulds (RF) distance^47^ is employed to assess the quality of the trees between the inferred trees and the acknowledgments.

And also the RF distance is used to measure the smallest distance between trees to see the better inferred trees. Table 2 summarizes the results of RF distances predicted by the ClustalW2. The minimum distances in each row are indicated in bold. The results inferred that the phylogenetic trees inferred from the BFO-GA resulting alignments has the smallest distances in five of eight databases. One of the common performances metric for measuring the quality of phylogenetic trees is RF distance metric. Only it may lack discriminatory power under various circumstances^48, 49^. This study provides preliminary evidences that BFO-GA may be safer, and more broad subject is required.

**Table 2:** RF Distances of the Inferred Phylogenetic Trees.

S.NO	Dataset		Algorithms				
			GA	ACO	ABC	PSO	BFO-GA
1	BaliBASE	RV 1	0.402339	0.399962	0.401872	0.398151	**0.394902**
2		RV 2	0.404595	0.407592	0.404414	0.407402	0.409055
3		RV 3	0.427078	0.42757	0.429009	0.429646	**0.424247**
4		RV 4	0.39619	0.399164	0.379741	0.396312	**0.370623**
5		RV 5	0.440182	0.448161	0.439905	0.438368	0.439634
6	Oxbench		0.409984	0.407611	0.405021	0.417541	0.410576
7	Prefab		0.417562	0.414324	0.417671	0.415848	**0.412834**
8	SABmark		0.399707	0.399491	0.395691	0.404367	**0.390916**

### Discussions

#### Discussion on the Multi-objectives of BFO-GA

The experiment has been carried out for 25 runs with 500 generations for 2%, 5%, 10%, 15% and 19% gap values respectively. Later all the iterations the average values are taken for 2,5,10, 15 and 19 percent gap values accordingly and the better values are identified at 19% gap. The results demonstrate that the proposed BFO-GA algorithm is more respectable among the other existing algorithms with respect to Affine and Variable Gap penalty Values. From these outcomes, it is understood that for the Similarity and Non-Gap Percentage objective values have been increased gradually, while the percentage of gap values have to increase. Accordingly the Gap penalties of Affine and Variable Gaps values have been decreased simultaneously when increasing the percentage of inserting gap value.

#### Efficiency on Performance Measures by the BFO-GA algorithm

The Sum of Pairs (SP) and the Total Column Score (TCS) are chosen as performance measure to compare the proposed algorithm with the existing algorithms. The mean value of 25 runs for 2%, 5%, 10%, 15% and 19% gap values indicates that the proposed algorithm provides better performance than the existing algorithms. In the beginning of the experiment iterations the SP and TCS value fluctuates in 5% and 10% and in later 15% and 19% iterations the SP and TCS values has increased. From all the iterations, it is noted that the proposed algorithm has best average results and it is found that for 19% gap penalty value better results are reached among all the iterations. For all the BAliBASE datasets the proposed algorithm provides more dependable outcomes with respect to affine and variable gap penalty values. Based on the experimental results and discussion, this research work concludes that the proposed BFO-GA algorithm can improve both the multi-objectives and performance measures than the existing algorithms.

## Conclusion and Future Enhancement

Today, the multiple sequence alignment problems are an unresolved issue for researchers. The alignment methods used to solve this problem should be habitually enhanced as they are important in the analysis of enormous data provided by next-generation sequencing and high-throughput experiments. The primary objective of this research study is to assess the evolutionary algorithms such as GA, ACO, ABC, PSO and exploring ways to further improve its execution to arrive at optimal solution. After careful analysis of the existing algorithms, this research work proposed BFO-GA algorithm to perform multiple sequence alignment and directs the result towards an optimal answer. The multi-objective optimization technique is used to resolve the problem which maximizes the similarity, non-gap percentage, and minimizes the value of gap penalty which goes to the Pareto - optimal result.

The statistical significance is computed to compare the significance of the proposed algorithm with other existing methods by using the Wilcoxon Matched-Pair Signed-Rank test. From the experimental results, it is exposed that the proposed BFO-GA algorithm outperforms the other existing algorithm in terms of all Multi-objectives and performance measures. And besides the proposed algorithm achieves good outcomes yet for low similarity of the sequences. The conserved blocks are not received, while performing the multiple sequence alignment. Hence it is concluded that they are not homologous sequences. Ultimately, the phylogenetic tree is constructed for the RV3 reference family in BaliBASE 3.0 by using the resulting MSA alignments provided by the proposed BFO-GA algorithm. Based on the RF distance values, it is inferred that the proposed algorithm achieves better results than the other methods.

In future the proposed algorithm can be blended or run with any other evolutionary algorithm to obtain the best optimal results. Different objectives may be innovated to find most excellent solutions of multiple sequence alignment and to get more conserved blocks. As well, this algorithm can be utilized for secondary and tertiary structure prediction of these successions.

## Electronic supplementary material

Supplementary Information
