# Supplementary material for: Bacterial Foraging Optimization –Genetic Algorithm for Multiple Sequence Alignment with Multi-Objectives
Source: Sci Rep. 2017 Aug 18;7:8833. doi: 10.1038/s41598-017-09499-1 (PMC5562892; doi:10.1038/s41598-017-09499-1)
Supplement: Supplementary file 1 — Supplementary Information [file 41598_2017_9499_MOESM1_ESM.pdf]

Bacterial Foraging Optimization –Genetic Algorithm for Multiple Sequence Alignment with Multi-Objectives

P.Manikandan[1], Dr. D. Ramyachitra[2],
[1]Research Scholar, [2]Assistant Professor,
Department of Computer Science, Bharathiar University, Coimbatore-641 046, India.
manimkn89@gmail.com[1], jaichitra1@yahoo.co.in.

Performance Measures of Optimization Algorithms and Phylogenetic Tree Construction

The Supplementary Table 1-2 shows the comparison of performance measures such as the Sum-of-Pairs (SP) and Total Column Score (TCS) for the proposed and existing algorithms (GA, ACO, ABC, PSO) and also with various online MSA tools such as T-Coffee, Muscle, K-Align, MAFFT and Clustal Omega. From these results, it is inferred that the proposed BFO-GA algorithm provides better results than the existing methods and tools. Also the Supplementary Fig. 1 shows the small tree containing a part of Operational Taxonomic Unit (OTUs) for the proposed and existing algorithms with respect to RV3 reference family in BaliBASE database.

Table.1 : Comparisons of Sum of Pairs (SP) scores for the existing and the proposed algorithm for the MSA datasets

Database	T-Coffee	Clustal Omega	Kalign	MUSCLE	MAFFT	GA	ACO	ABC	PSO	**BFO-GA**
Balibase	54	55	55	59	61	63	70	80	83	**90**
Sabmark	57	58	59	61	62	64	74	82	82	**90**
Prefab	57	52	51	55	59	60	65	80	76	**88**
Oxbench	50	49	51	55	58	61	74	83	86	**90**

Table.2 : Comparisons of Total Column Score (TCS) scores for the existing and the proposed algorithm for the MSA datasets

Database	T-Coffee	Clustal Omega	Kalign	MUSCLE	MAFFT	GA	ACO	ABC	PSO	**BFO-GA**
Balibase	42	43	45	49	53	58	58	56	67	**77**
Sabmark	43	48	52	53	55	57	56	61	55	**79**
Prefab	51	49	50	53	54	56	59	64	59	**75**
Oxbench	48	46	50	52	54	56	59	67	56	**75**

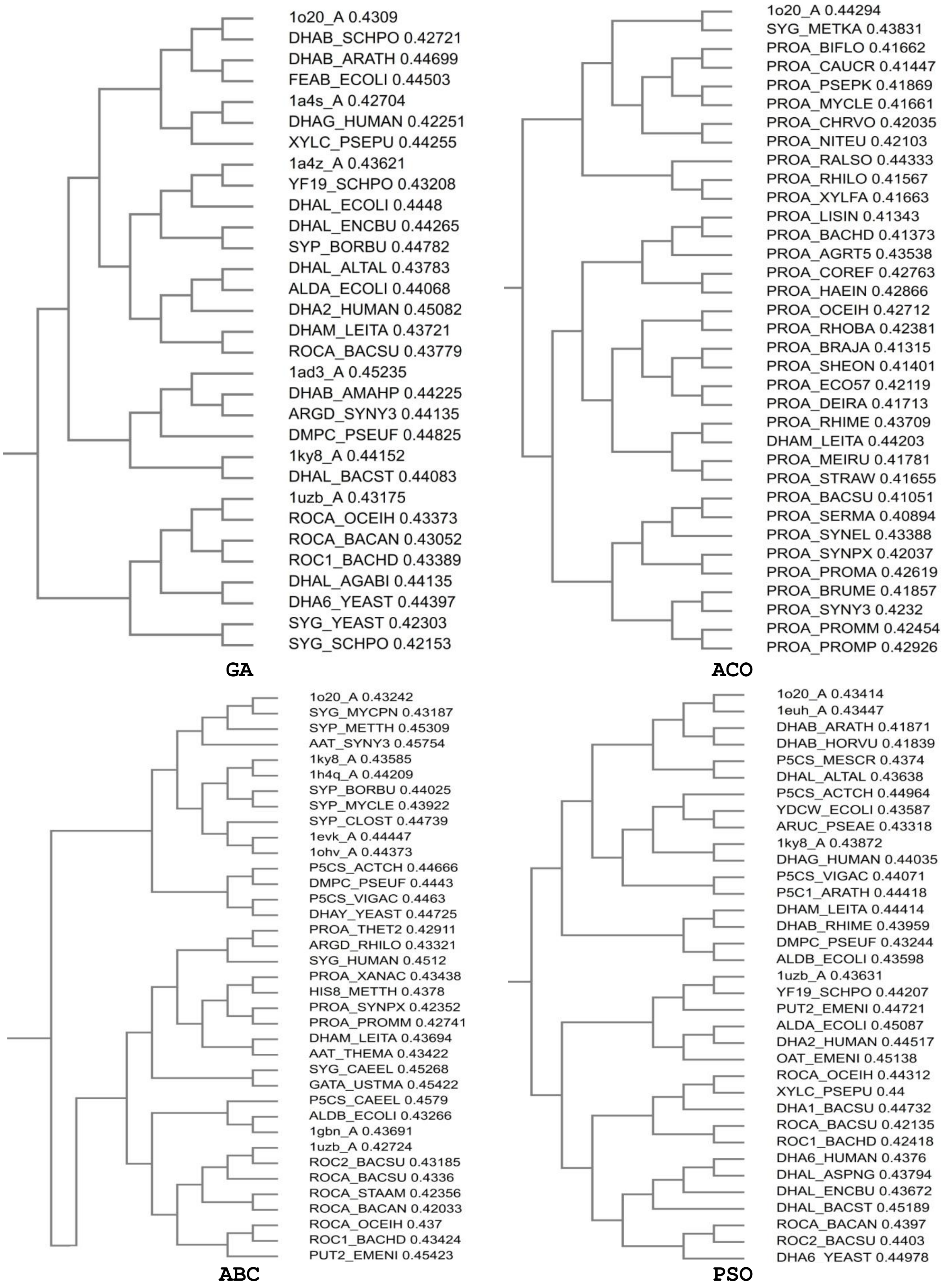
1o20_A 0.4309
DHAB_SCHPO 0.42721
DHAB_ARATH 0.44699
FEAB_ECOLI 0.44503
1a4s_A 0.42704
DHAG_HUMAN 0.42251
XYLC_PSEPU 0.44255
1a4z_A 0.43621
YF19_SCHPO 0.43208
DHAL_ECOLI 0.4448
DHAL_ENCBU 0.44265
SYP_BORBU 0.44782
DHAL_ALTAL 0.43783
ALDA_ECOLI 0.44068
DHA2_HUMAN 0.45082
DHAM_LEITA 0.43721
ROCA_BACSU 0.43779
1ad3_A 0.45235
DHAB_AMAHP 0.44225
ARGD_SYNY3 0.44135
DMPC_PSEUF 0.44825
1ky8_A 0.44152
DHAL_BACST 0.44083
1uzb_A 0.43175
ROCA_OCEIH 0.43373
ROCA_BACAN 0.43052
ROC1_BACHD 0.43389
DHAL_AGABI 0.44135
DHA6_YEAST 0.44397
SYG_YEAST 0.42303
SYG_SCHPO 0.42153
GA
1o20_A 0.44294
SYG_METKA 0.43831
PROA_BIFLO 0.41662
PROA_CAUCR 0.41447
PROA_PSEPK 0.41869
PROA_MYCLE 0.41661
PROA_CHRVO 0.42035
PROA_NITEU 0.42103
PROA_RALSO 0.44333
PROA_RHILO 0.41567
PROA_XYLFA 0.41663
PROA_LISIN 0.41343
PROA_BACHD 0.41373
PROA_AGRT5 0.43538
PROA_COREF 0.42763
PROA_HAEIN 0.42866
PROA_OCEIH 0.42712
PROA_RHOBA 0.42381
PROA_BRAJA 0.41315
PROA_SHEON 0.41401
PROA_ECO57 0.42119
PROA_DEIRA 0.41713
PROA_RHIME 0.43709
DHAM_LEITA 0.44203
PROA_MEIRU 0.41781
PROA_STRAW 0.41655
PROA_BACSU 0.41051
PROA_SERMA 0.40894
PROA_SYNEL 0.43388
PROA_SYNPX 0.42037
PROA_PROMA 0.42619
PROA_BRUME 0.41857
PROA_SYNY3 0.4232
PROA_PROMM 0.42454
PROA_PROMP 0.42926
ACO
1o20_A 0.43242
SYG_MYCPN 0.43187
SYP_METTH 0.45309
AAT_SYNY3 0.45754
1ky8_A 0.43585
1h4q_A 0.44209
SYP_BORBU 0.44025
SYP_MYCLE 0.43922
SYP_CLOST 0.44739
1evk_A 0.44447
1ohv_A 0.44373
P5CS_ACTCH 0.44666
DMPC_PSEUF 0.4443
P5CS_VIGAC 0.4463
DHAY_YEAST 0.44725
PROA_THET2 0.42911
ARGD_RHILO 0.43321
SYG_HUMAN 0.4512
PROA_XANAC 0.43438
HIS8_METTH 0.4378
PROA_SYNPX 0.42352
PROA_PROMM 0.42741
DHAM_LEITA 0.43694
AAT_THEMA 0.43422
SYG_CAEEL 0.45268
GATA_USTMA 0.45422
P5CS_CAEEL 0.4579
ALDB_ECOLI 0.43266
1gbn_A 0.43691
1uzb_A 0.42724
ROC2_BACSU 0.43185
ROCA_BACSU 0.4336
ROCA_STAAM 0.42356
ROCA_BACAN 0.42033
ROCA_OCEIH 0.437
ROC1_BACHD 0.43424
PUT2_EMENI 0.45423
ABC
1o20_A 0.43414
1euh_A 0.43447
DHAB_ARATH 0.41871
DHAB_HORVU 0.41839
P5CS_MESCR 0.4374
DHAL_ALTAL 0.43638
P5CS_ACTCH 0.44964
YDCW_ECOLI 0.43587
ARUC_PSEAE 0.43318
1ky8_A 0.43872
DHAG_HUMAN 0.44035
P5CS_VIGAC 0.44071
P5C1_ARATH 0.44418
DHAM_LEITA 0.44414
DHAB_RHIME 0.43959
DMPC_PSEUF 0.43244
ALDB_ECOLI 0.43598
1uzb_A 0.43631
YF19_SCHPO 0.44207
PUT2_EMENI 0.44721
ALDA_ECOLI 0.45087
DHA2_HUMAN 0.44517
OAT_EMENI 0.45138
ROCA_OCEIH 0.44312
XYLC_PSEPU 0.44
DHA1_BACSU 0.44732
ROCA_BACSU 0.42135
ROC1_BACHD 0.42418
DHA6_HUMAN 0.4376
DHAL_ASPNG 0.43794
DHAL_ENCBU 0.43672
DHAL_BACST 0.45189
ROCA_BACAN 0.4397
ROC2_BACSU 0.4403
DHA6_YEAST 0.44978
PSO

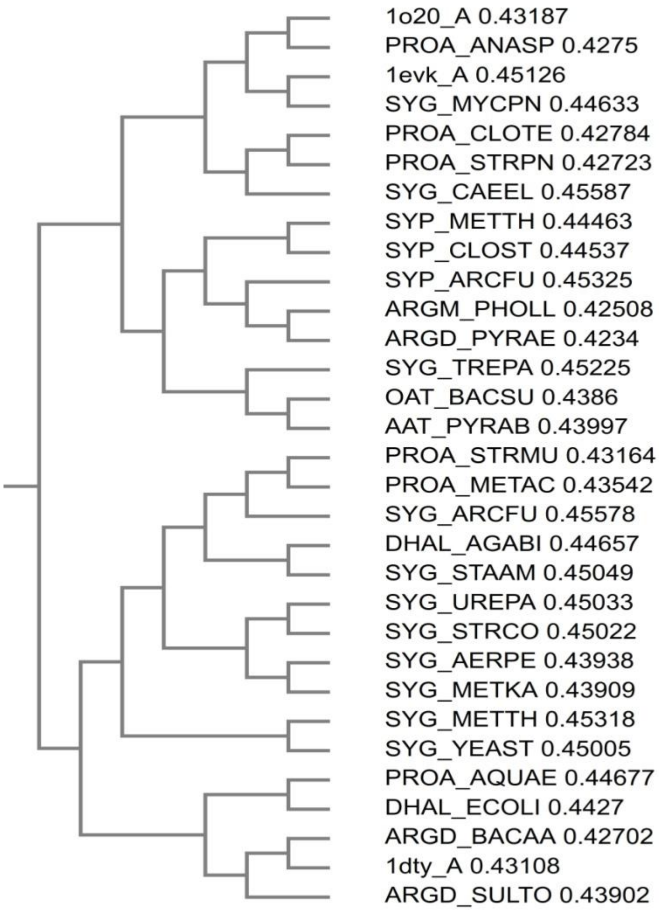

BFO-GA

Fig. 1: Phylogenetic Trees of RV 3 reference family in BaliBASE 3.0 Dataset
